# The Rho family GEF FARP2 is activated by aPKCι to control tight junction formation and polarity

**DOI:** 10.1242/jcs.223743

**Published:** 2019-04-25

**Authors:** Ahmed Elbediwy, Yixiao Zhang, Mathias Cobbaut, Philippe Riou, Ray S. Tan, Selene K. Roberts, Chris Tynan, Roger George, Svend Kjaer, Marisa L. Martin-Fernandez, Barry J. Thompson, Neil Q. McDonald, Peter J. Parker

**Affiliations:** 1 Epithelial Biology Laboratory, Francis Crick Institute, 1 Midland Road, London NE1 1AT, UK; 2 Protein Phosphorylation Laboratory, Francis Crick Institute, 1 Midland Road, London NE1 1AT, UK; 3 Structural Biology Team, Francis Crick Institute, 1 Midland Road, London NE1 1AT, UK; 4 Signalling and Structural Biology Laboratory, Francis Crick Institute, 1 Midland Road, London NE1 1AT, UK; 5 School of Cancer and Pharmaceutical Sciences, King's College London, Guy's Campus, London SE1 1UL, UK; 6 Central Laser Facility, STFC Rutherford Appleton Laboratory, Harwell Oxford, Didcot, Oxford OX11 0QX, UK

**Keywords:** Cdc42, FARP, Atypical protein kinase C, Polarity

## Abstract

The elaboration of polarity is central to organismal development and to the maintenance of functional epithelia. Among the controls determining polarity are the PAR proteins, PAR6, aPKCι and PAR3, regulating both known and unknown effectors. Here, we identify FARP2 as a ‘RIPR’ motif-dependent partner and substrate of aPKCι that is required for efficient polarisation and junction formation. Binding is conferred by a FERM/FA domain–kinase domain interaction and detachment promoted by aPKCι-dependent phosphorylation. FARP2 is shown to promote GTP loading of Cdc42, which is consistent with it being involved in upstream regulation of the polarising PAR6–aPKCι complex. However, we show that aPKCι acts to promote the localised activity of FARP2 through phosphorylation. We conclude that this aPKCι−FARP2 complex formation acts as a positive feedback control to drive polarisation through aPKCι and other Cdc42 effectors.

This article has an associated First Person interview with the first author of the paper.

## Abstract (precis)

**Summary:** The exchange factor FARP2 is identified as a polarity-determining protein that is a partner and substrate of aPKCι. Phosphorylation by aPKCι is shown to control the localised action of FARP2 on Cdc42.

## INTRODUCTION

Atypical protein kinase Cs (PKC), aPKCζ and aPKCι, are serine/threonine specific protein kinases that form a distinctive subset of PKC proteins with characteristic regulatory inputs, outputs and pharmacology (for a review, see Parker et al., 2014). The most well-characterised physiological role relates to aPKCι and its requirement for determining asymmetric/polarised cellular behaviours (reviewed in Chen and Zhang, 2013; Suzuki and Ohno, 2006). This was initially established in *Caenorhabditis elegans* (Tabuse et al., 1998) where the aPKC orthologue, along with other PAR proteins, have been shown to play critical roles in cell polarisation; the same conserved modules, aPKC, PAR6 and PAR3, were subsequently shown to operate in mammals (note in mammals there are several PAR6 and PAR3 family proteins) (Joberty et al., 2000).

The direct interaction of aPKCι with regulatory proteins and substrates is a particular feature of its action. In *C. elegans* there is a dynamic cycling between highly localised PAR3-containing aPKCι complexes (inactive) and dispersed Cdc42 containing complexes (active) (Rodriguez et al., 2017); the inactivity being determined by interaction of the CR3 region of PAR3 with the substrate-binding pocket of aPKCι (Soriano et al., 2016). Mutation of the aPKCι RIPR partner interaction motif, as seen rarely but repeatedly in cancers, leads to a failure of the mutant protein to support normal polarisation (Linch et al., 2013). In pathophysiological states, aPKCι hyperactivation through Ras-dependent mechanisms can also drive a loss of polarity (Linch et al., 2014); such aPKC hyperactivation has been reported to overcome contact inhibition through Hippo/Yap signalling (Archibald et al., 2015). This suppression of polarity-dependent growth inhibition is consistent with a role in tumorigenesis as seen in an inducible lung model of Ras-dependent tumour formation (Regala et al., 2009).

FERM, RhoGEF and pleckstrin domain-containing proteins (FARPs) are guanine nucleotide exchange factors (GEFs) for Rho family proteins (Kubo et al., 2002; Ni et al., 2003; Toyofuku et al., 2005), and FARP2 is identified here as a protein partner in an aPKCι interactome screen. FARP2 is shown to act as a GEF for the upstream polarity regulator Cdc42 (Noda et al., 2001); however, we demonstrate that FARP2 also acts downstream of aPKCι, where it controls polarity. The aPKCι–FARP2 module thus comprises a novel positive feedback control acting to regulate polarity through its own assembly and turnover.

## RESULTS AND DISCUSSION

### aPKCι interacts with and phosphorylates FARP proteins

**Fig. 1.**
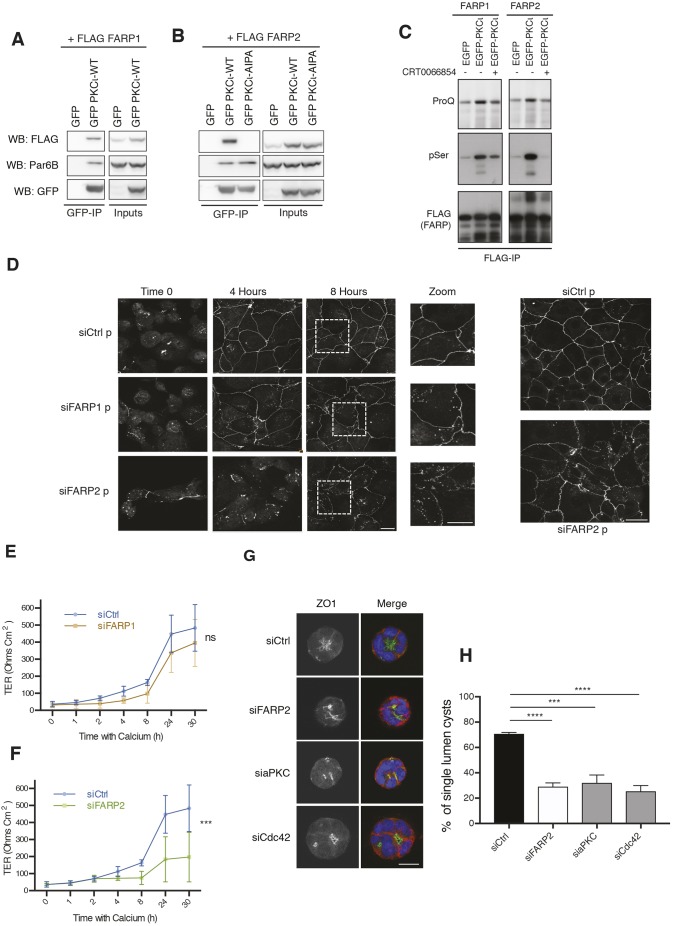
**FARP2 is a RIPR-dependent substrate of aPKCɩ that is responsible for maintaining tight junctions and polarity.** (A,B) FARP1 and FARP2 co-precipitate with aPKC. HCT116 cells were co-transfected with plasmids expressing FLAG-tagged FARP1 (A) or FARP2 (B) and GFP, GFP-tagged aPKCι or GFP-tagged aPKCι containing a RIPR to AIPA mutation (R480A/R483A). Immunoprecipitates were analysed with the indicated antibodies. Images are of representative blots of *n*=3. (C) GFP–PKCι phosphorylates FARP1 and FARP2 in cells. HCT116 cells were co-transfected with plasmids expressing FLAG-tagged FARP1 or FARP2, and GFP or GFP-tagged aPKCι. Immunoprecipitates (IP) were analysed via ProQ diamond staining or with the indicated antibodies. (D) FARP2 and not FARP1 is involved in junctional establishment after Ca^2+^ switch. Caco-2 cells were subjected to siRNA treatment (p represents the use of ON-TARGETplus SMARTpool siRNA, Dharmacon), processed for Ca^2+^ switch immunofluorescence and stained for the junctional marker ZO-1. A representative example of *n*=3 experiments with six coverslips per immunofluorescence experiment is shown. (E) FARP1 depletion has no effect on junctional permeability as indicated by a Ca^2+^ switch TER assay. A representative example of *n*=3 experiments is shown. (F) FARP2 depletion has a substantial effect on junctional permeability as indicated by a Ca^2+^ switch transepithelial assay. A representative example of *n*=3 experiments with six samples per experiment is shown. (G) 3D lumen formation in a CaCo2 model is disturbed upon knockdown of either FARP2, Cdc42 or PKCι. CaCo2 cells were grown on a Matrigel-coated surface as described in the Materials and Methods. Cysts were stained for ZO-1 (green), F-actin (red) as indicated and Hoechst 33342 (stained according to manufacturer's instructions; Sigma-Aldrich) (blue). (H) Quantification of the proportion of single lumen cysts for experiments as in G. *n*≥100 cysts were counted per experiment. Results are mean±s.d. ns, not significant (*P*>0.05); ***P≤0.001; ****P≤0.0001 (unpaired *t*-test). siCtrl, control siRNA. Scale bars: 20 μm.

A proteomics screen for endogenously expressed proteins associating with aPKCι in HCT116 cells revealed that FARP2 is an aPKCι interactor (Fig. S1A). We validated the interaction of aPKCι with FARPs by co-expression with aPKCι and immunoprecipitation (antisera to the endogenous protein was not effective for native aPKCι recovery). aPKCι efficiently binds to both FARP1 and FARP2 (Fig. 1A,B). Complex formation with FARP2 was corroborated in cells employing a fluorescence resonance energy transfer–fluorescence-lifetime imaging microscopy (FRET-FLIM)-based approach (Fig. S1B). Co-expression with aPKCι revealed an increase in overall and PKC-mediated phosphorylation of FARP1/2, as revealed by ProQ Diamond staining and phosphorylated serine (pSer) PKC substrate immunostaining, respectively (Fig. 1C). Increased phosphorylation of FARP1/2 was inhibited by a pre-incubation with the aPKCι specific inhibitor CRT0066854 (Kjaer et al., 2013), indicating that both FARP proteins are phosphorylated under aPKCι control (Fig. 1C).

Through co-immunoprecipitation, we established that FARP2 is a RIPR motif-dependent partner (Fig. 1C) (Linch et al., 2013). Furthermore, through deletion mapping, we found that aPKCι interacts with FARP2 via its FERM and FERM-adjacent (FA) domains (Fig. S1C), a conserved domain pair retained in various proteins (see Moleirinho et al., 2013). Interaction with FARP2 could be reproduced when using the aPKCι kinase domain alone. Furthermore a stoichiometric complex of the GST–kinase domain and the FARP2 FERM-FA domains could be isolated from sf9 cells, indicating that these regions are sufficient for interaction, although not stable to subsequent gel filtration once cleaved from the GST fusion partner (Fig. S1D).

### FARP2 but not FARP1 is required for polarity in Caco2 cells

Using ZO-1 (also known as TJP1) staining as a proxy for intact, polarised cell–cell contacts, we found impaired establishment of cell–cell junctions in cells depleted of FARP2 (siFARP2), but not of FARP1 (siFAPR1) (Fig. 1D; see knockdown Fig. S1E). This phenocopies what is seen upon Cdc42 and aPKCι depletion (Figs S2A and S3B). Consistent with this altered behaviour after knockdown of FARP2, aPKC was also lost from cell–cell junctions (Fig. S2B). Furthermore, only FARP2 depletion resulted in a loss of trans-epithelial resistance (TER), a functional marker of intact cell–cell contacts (Fig. 1E,F). In a direct assessment of polarity, knockdown of FARP2 also phenocopied the knockdown of either Cdc42 or aPKCι in a Caco 3D lumen formation assay (Durgan et al., 2011), indicative of a loss of polarised morphogenesis (Fig. 1G,H).

### FARP2 is required for efficient initiation of junction formation

**Fig. 2.**
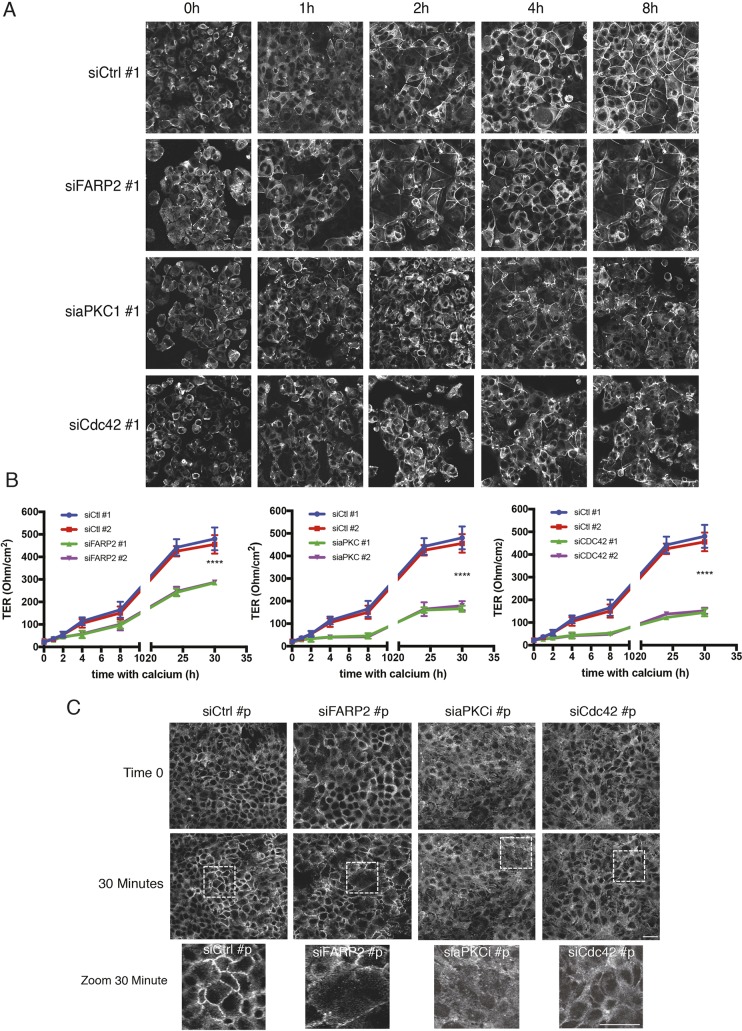
**FARP2 is required for efficient initiation of junction formation.** (A) Individual siRNA oligonucleotides directed at FARP2 cause severe disruption of ZO-1 during junction establishment (see also Fig. S2). A representative example or *n*=3 with six samples per experiment is shown. (B) FARP2, aPKC and Cdc42 siRNA deconvolution in a Ca^2+^ switch assay. The TER is severely disrupted, indicative of loss of junctional integrity. A representative example of *n*=3 experiments with five samples per experiment is shown. (C) *De novo* junction formation in EGF-stimulated A431 cells. Pooled siRNA (denoted by p, siGenome Pools) directed at FARP2, aPKC or Cdc42 results in junctional impairment, indicated by the loss of integrity of ZO-1. A representative example or *n*=3 with six samples per experiment is shown. Results in B are mean±s.d. ****P≤0.0001 (unpaired *t*-test). siCtrl, control siRNA. Scale bars: 20 μm.

To assess whether FARP2 also had a role in junctional initiation, cells were subjected to a Ca^2+^ switch (Elbediwy et al., 2012). Depletion of FARP2 prevented proper junctional establishment, as evident through the disorder of the marker ZO-1; this was seen prominently at 8 h post Ca^2+^ re-addition, phenocopying the effects of aPKCι and Cdc42 knockdown (Fig. 2A). Following depletion of FARP2 with a validated siRNA, we found that the TER was significantly reduced (∼40%), which is a similar level of reduction to what is seen upon aPKCι or Cdc42 depletion (Fig. 2B). These effects were also observed with a second FARP2 siRNA (Fig. S3A).

To assess the penetrance of this dependence on FARP2 for *de novo* junction formation, we employed A431 cells. When these cells are serum-starved, ZO-1 is lost from cell–cell contacts and upon addition of EGF, ZO-1 relocalises in a time-dependent fashion as tight junctions (TJs) re-form (Van Itallie et al., 1995). We depleted FARP2 and assessed ZO-1 localisation at time 0 and 30 min post EGF addition. We found that the normal coherent localisation of ZO-1 became severely fragmented upon depletion of FARP2, further validating a role for FARP2 in junction establishment (Fig. 2C). By using individual siRNAs directed at FARP2 in Caco-2 cells, we also observed a disruption of ZO-1 localisation (Fig. S3B) and a drop in TER, albeit to a lesser extent than observed in the establishment assay (Fig. S3C). This indicates that FARP2 is involved primarily in junctional establishment but also to some extent in their maturation and/or maintenance. It is surmised that removal of the GEF results in aberrant signalling, disrupting cell–cell contacts.

### FARP2 acts as a Cdc42-GEF in Caco2 cells

**Fig. 3.**
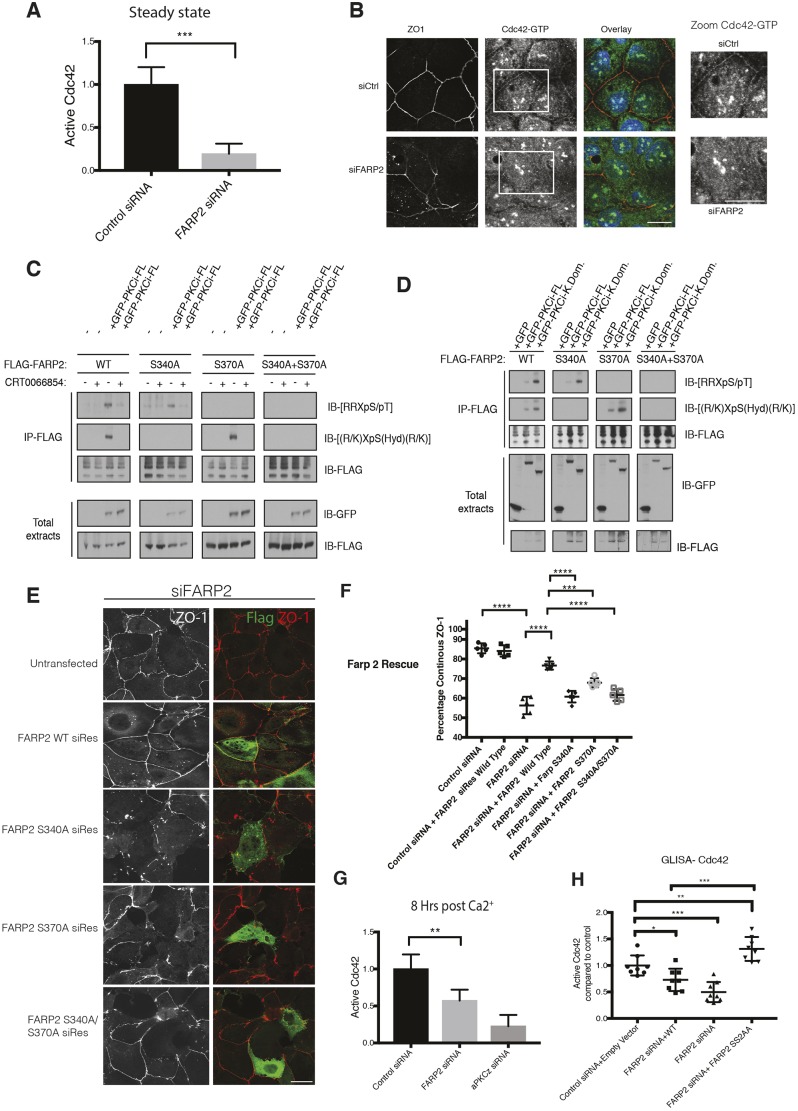
**Molecular function of FARP2 and the effect of aPKCι-mediated phosphorylation.** (A) G-LISA assay assessing the levels of active Cdc42 in CaCo2 cells transfected with either control siRNA (siCtrl) or siRNA targeting FARP2; *n*=3. (B) FARP2 depletion impairs localisation of Cdc42-GTP and ZO1 at cell–cell junctions. A representative example of *n*=2 experiments with five samples per experiment. (C) FARP2 is phosphorylated by aPKCι. FARP2 WT or mutants were expressed in HCT116 cells with or without aPKCι and immunoprecipitated (IP). Phosphorylation at S340 and S370 was assessed using antibodies that recognise the sequence context of each site. The use of aPKCι-specific inhibitor CRT0066854 (10 µM, 60 min) confirmed aPKCι-mediated phosphorylation. Representative blots of *n*=2 experiments are shown. (D) Active aPKCι phosphorylates FARP2 without requiring its regulatory region. HCT116 cells were co-transfected with WT FARP2 or mutants as indicated with or without aPKCι or its kinase domain (K.Dom.). FARP2 was immunoprecipitated, and phosphorylation at S340 and S370 was assessed as in C. Representative blots of *n*=2 experiments are shown. (E,F) Mutation of the S340 and S370 phosphorylation sites in FARP2 prevents siRNA-resistant FARP2 from rescuing the altered ZO-1 localisation phenotype observed upon FARP2 depletion. The location of ZO-1 is indicated (left panels; red in merge) alongside the GFP expression (right panels; green in merge). A representative example of *n*=3 experiments with six samples per experiment is shown. A quantitative analysis is shown in the histogram (F) as indicated for the different rescue constructs. (G) Levels of active Cdc42 during a Ca^2+^ switch. The effects of FARP2 or aPKCι knockdown at 8 h post Ca^2+^ re-addition result in severe depletion of Cdc42-GTP. A representative example of *n*=3 experiments with six samples per experiment is shown. (H) Levels of active Cdc42 are rescued by both the WT and mutant constructs. A representative example of *n*=2 experiments with eight samples per experiment is shown. Results are mean±s.d. *P≤0.05; **P≤0.01; ***P≤0.001; ****P≤0.0001 (unpaired *t*-test). Scale bars: 20 μm.

There are differences observed with respect to the G-protein specificity of FARP proteins (see Kubo et al., 2002; Miyamoto et al., 2003). To assess whether FARP2 acts through its GEF activity for junction establishment we used a G-LISA activation assay. FARP2 depletion significantly affected the levels of active Cdc42 (Fig. 3A), while having no significant effect on Rac1, Rac2 or Rac3 (Fig. S4A). We further assessed this with an anti-Cdc42-GTP antibody (Elbediwy et al., 2012). In control cells, Cdc42-GTP localises in part to the TJs, while on FARP2 depletion, its junctional localisation is disrupted (Fig. 3B), suggesting that FARP2 is indeed a GEF for Cdc42 in this model. This is consistent with the notion that FARP2 acts to increase Cdc42-GTP levels and hence triggers aPKCι activation acting through PAR6 (Noda et al., 2001), and that all three proteins are required for the initiation and maintenance of polarity (see Chen and Zhang, 2013; Suzuki and Ohno, 2006). However, it transpires this is a more complex feedback control pathway as indicated by the influence of aPKCι on FARP2.

### aPKCι phosphorylates sites in the FARP2 FERM-FA domains to control polarity

*In silico* analysis and *in vitro* peptide screening with recombinant aPKCι identified two candidate phosphorylation sites in FARP2 that are also conserved in FARP1 and partially in two EPB41 family members (Fig. S4B); both FARP2 sites, S340 and S370, are located in the FERM-FA domain where aPKCι interacts. To test whether these sites were phosphorylated by aPKCι in cells, we co-expressed aPKCι with wild-type FARP2 (FARP2 WT) or FARP2 with phosphorylation-resistant mutants (S340A/S370A mutations). Analysis via immunoblotting with antibodies that recognise the motifs surrounding either S340 or S370, revealed that these sites could be targeted by aPKCι in a manner inhibited by the selective drug CRT0066854 (Kjaer et al., 2013) (Fig. 3C). Consistent with activation-dependent phosphorylation, the kinase domain was more efficient than the full-length protein in supporting this phosphorylation, whilst retaining the same pattern of specificity for these two sites (Fig. 3C,D). The total absence of detectable phosphorylation in the double S340A/S370A mutant demonstrates that these two sites are the dominant aPKCι phosphorylation sites recognised.

To establish the influence of aPKCι-mediated phosphorylation of FARP2 on junction integrity, we knocked down FARP2 and assessed rescue with siRNA resistant mutants. Upon FARP2 depletion, ZO-1 was perturbed as expected; this phenotype was reversed by re-expressing a WT FARP2 siRNA resistant mutant. Expression of FARP2 does not influence ZO-1 in a control siRNA background (Fig. S4C). Notably, expressing siRNA-resistant forms of the non-phosphorylatable mutants of FARP2 fails to rescue junctional disruption, indicating that aPKCι-dependent phosphorylation of FARP2 is essential for junctional integrity (Fig. 3E,F).

To assess whether phosphorylation of FARP2 acted to control catalytic activity of FARP2 directly, we overexpressed FARP2 WT or the S340A/S370A double-mutant together with aPKCι and assessed active Cdc42 levels using a PAK1-PBD pulldown assay. Both FARP2 constructs led to an increase in Cdc42-GTP and this was not influenced by aPKCι inhibition (Fig. S3G). We also sought to rescue levels of active Cdc42 by expressing either siRNA resistant WT or mutant FARP2 in the context of endogenous FARP2 knockdown in a GLISA assay; expression of WT FARP2 rescued the levels of active Cdc42 (Fig. 3H) and we observed an even stronger recovery of Cdc42-GTP with the non-phosphorylatable mutant. The evidence is compelling that, biochemically, phosphorylation by aPKCι does not act directly on the GEF activity of FARP2. It is not clear why the S340A/S370A FARP2 mutant might be more effective than WT FARP2 in elevating steady-state levels of Cdc42-GTP. This might reflect distinct localisation of this ectopically expressed mutant (aligned with the conclusions relating to aPKCι action, see below), perhaps changing its juxtaposition to Cdc42 GAPs.

Since ectopic expression typically compromises compartmentalisation and there was a requirement for FARP2 phosphorylation in initiating efficient junction formation, phenocopying aPKCι loss, we determined whether GTP loading of Cdc42 was influenced by endogenous aPKCι. We assessed the levels of active Cdc42 following a Ca^2+^ switch and the associated effects of FARP2 or aPKCι knockdown. At 8 h post Ca^2+^ re-addition, when the loss of polarisation was evident (see above), the level of GTP-bound Cdc42 was significantly reduced upon depletion of either FARP2 or aPKCι (Fig. 3G). It appears that aPKCι exerts positive-feedback control on its upstream regulator Cdc42 but, as determined by ectopic co-expression experiments, this is not a simple biochemical consequence of phosphorylation of FARP2 by aPKCι. The effects on the endogenous FARP2 compared to the lack of effect on the properties of the ectopic FARP2 suggested that localisation was likely to be an important factor in this pathway.

When FARP2 phosphorylation is blocked through aPKCι inhibition, there was a stabilisation of the FARP2–aPKCι complex (Fig. S4D). Similarly co-expression with a catalytically inactive aPKCι led to an increased recovery of the complex (Fig. S4E). Finally, consistent with the site mapping, stabilisation of the complex was also seen upon mutation of the two identified target sites, the effect being dominated by the S340 site and showing no influence from aPKCι inhibition (Fig. S4F). This demonstrates that phosphorylation of FARP2, which is required for its effects on polarisation, is associated with turnover of its complex with aPKCι. This led us to conclude that the release of FARP2 from the aPKCι complex and its subsequent transition to or function at junctional compartments (see Fig. 3E,F) might be critical to aPKCι action. Since loss of FARP2 function disrupts ZO-1 localisation, we cannot determine formally whether this is causal or consequential; however, the weight of evidence on the requirements, the localisation of active Cdc42 at junctions and the notable precedent of PAR3 behaviour (Soriano et al., 2016) suggest that FARP2 is released to act at the junctional compartment following aPKCι-mediated phosphorylation. Monitoring the expression levels of aPKCι and the polarity marker Par6 we find no effect upon FARP2 siRNA treatment, while the localisation of aPKCι and the polarity marker ezrin is severely affected (see Fig. S2B,C). This was confirmed by the use of CRT0066854 and expression of the WT form of FARP2. This construct fails to rescue the polarity phenotype seen upon aPKCι inhibition as it requires the activity of aPKCι (Fig. S4H).

**Fig. 4.**
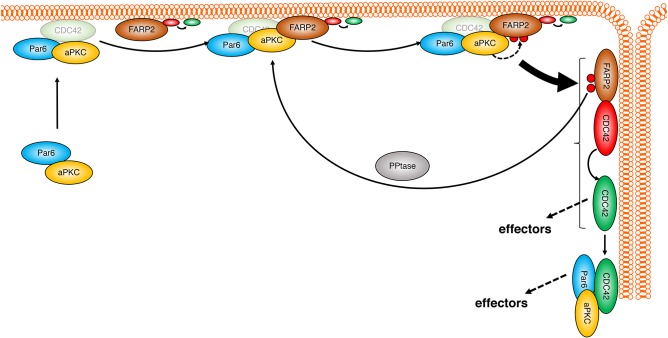
**Hypothetical model of a feedback activation mechanism for aPKC during junction establishment and maintenance.** FARP2 associates with aPKC via a kinase domain RIPR-motif–FARP2FERM-FA interaction. Phosphorylation of FARP2 at S340 and S370 (red circles; dashed arrow) in the FA domain results in dissociation of the complex and promotes localised function of FARP2 at the junctions (indicated by the curly bracket), where it activates Cdc42. Activated Cdc42 (ovals with red to green transition; Ccd42 as a possible partner in membrane-associated aPKC–Par6 complexes is depicted in a faded shade) can activate downstream effectors such as aPKC containing complexes. Maintained aPKC activity results in continuous FARP2 phosphorylation, resulting in a positive-feedback cycle necessary to initiate and maintain junctions. FARP2 is also active independently of phosphorylation as depicted in the model. PPtase, phosphatase mediating dephosphorylation of FARP2.

In conclusion, as illustrated in Fig. 4, we identify FARP2 as a novel substrate of aPKCι and show that it is responsible for maintaining Cdc42-GTP levels under polarising conditions in the Caco2 model. Loss of any elements of this pathway compromises polarisation. As an effector of Cdc42, aPKCι, acting with FARP2, therefore appears to act as an amplifier. Active Cdc42 can activate a variety of additional downstream targets (reviewed in Etienne-Manneville, 2004); hence, this regulatory module confers a positive-feedback mechanism in which a FARP2–Cdc42 complex will not only activate Par6–aPKCι complexes, but also drive effector functions through additional Cdc42 downstream targets independently of aPKCι that are predicted to be effective in distinct junctional compartment(s). Although the exact molecular implications and the spatiotemporal importance of this complex assembly *in vivo* have yet to be elaborated, it is evident that this module is required for effective junction formation and maintenance.

## MATERIALS AND METHODS

### Cell culture, antibodies and chemicals

For cell line authentication, cell lines were mycoplasma screened and short tandem repeat (STR) profiled. The STR profile was cross referenced back to any available published profile for the cell line in question. If there was no published profile available, it was checked against the Cell Services STP database of the Francis Crick Institute, London, UK. HCT116 and Caco-2 cells were cultured as previously described (Linch et al., 2013; Soriano et al., 2016). A431 cell experiments were performed as previously described (Elbediwy et al., 2012; Van Itallie et al., 1995). Briefly, A431 cells were serum starved for 24 h before being stimulated with EGF at a final concentration of 100 ng/ml for the time points specified before being fixed.

Reagents used in this study include: ProQ diamond stain (Thermo Fisher Scientific), antibodies against FLAG M2 (Sigma, F3165; 1:1000), PAR6B (Santa Cruz Biotechnology, sc, H-64, 1:500), FARP1 (Santa Cruz Biotechnology, sc-74927, K-20, 1:250), FARP2 (Santa Cruz Biotechnology, sc-390744, H-9, 1:250), aPKC (BD Biosciences C-20, western blotting at 1:1000, immunofluorescence at 1:250) and GFP (FL, 1:1000) antibodies (Santa Cruz Biotechnology), phospho-PKA Substrate (RRXS*/T*) (100G7E, 1:1000) and phospho-(Ser) PKC substrate antibody (1:1000, Cell Signaling Technology), aPKC antibody (Cat number: 610608, 1:1000) (BD Transduction Laboratories), GAPDH antibody (MAB374, Millipore; 1:5000), HRP-conjugated secondary antibody (GE Healthcare), and ZO-1 (33-9100, Invitrogen, 1:400).

3D cultures were generated using CaCo2 cells. Briefly, cells were seeded and reverse transfected with siRNA, left for 3 days then seeded in eight-well chambers pre-coated with a 80% Matrigel and 20% collagen mix (Thermo Fisher Scientific, BD and Amsbio). Cells were resuspended at 8000 cells/ml in medium containing 2% Matrigel. Medium was replenished at day 6 and cells were fixed at day 8 with 10% formalin and processed for immunofluorescence as previously described (Elbediwy et al., 2012).

### Oligonucleotides, plasmids and cloning

For siRNA-mediated knockdown, oligonucleotides were obtained from (GE Dharmacon). The following siRNAs were used: siCTL 1, 5′-UAAGGCUAUGAAGAGAUAC-3′; siCTL 2, 5′-UAGCGACUAAACACAUCAA-3′; siFARP2-01, 5′- GAACAUACCUCAAGGAUUU-3′; siFARP2-02, GATTTGGCTTGAACCTAT-3′; aPKC#1, 5′-GGGUACAGACAGAGAAGCAUU-3′; aPKC#2, 5′- GUGUUUGAGCAGGCAUCCAUU-3′; Cdc42-01, 5′-CGGAAUAUGUACCGACUGU-3′; and Cdc42-02, 5′-GAUGACCCCUCUACUAUUG-3′. C-terminal FLAG–Myc-tagged FARP1 (RC208329) and FARP2 (RC216784) human cDNA constructs were purchased from OriGene. Sequencing revealed that both plasmids contain mutations different from the WT sequences. Therefore, site-directed mutagenesis (Quikchange, Agilent) was employed to obtain WT sequences (FARP1 Y644H FW, 5′-CTCACCTGTGGAAGCAGCGAGGCCTTG-3′; FARP2 P375S FW, 5′-CAAGACCCACACGTCCGTTCGAGCTCTG-3′), subsequently S340 or S370 mutations were introduced via Quikchange (FARP2 S340 FW: 5′-CAGCCGGGGCTCCGCCTTCAGATACAGT-3′; S370 FW: 5′-CGGACGTGTGGGTCTTGGCGTGCCTTCTTTCATATG-3′). siRNA-resistant mutants of FARP2 were also obtained via Quikchange (nt, 1644A>T, 1647A>T; FW 5′-GAGATTCTCGCTACAGAACGGACTTACCTCAAGGATTTAGAAGT-3′). The FARP2 FERM-FA domain was cloned in pCMV6-Entry (OriGene) using the following primers: Farp2-FERMFA-Fw, 5′-GATATAGCGATCGCCATGGGGGAGATAGAA-3′; and Farp2-FERMFA-Rv, 5′-CGATATACGCGTAGGAGTCCTCAATCCCTC-3′. aPKC constructs used in this paper have been described elsewhere (Linch et al., 2013). To express the FARP2 FERM-FA and aPKCι kinase domains in sf21 insect cells, the FERM-FA domain was cloned in pBacPAK-His3 via in-fusion (Takara) using the following primers: FW, 5′-ACCATCACGGGTCGACACAAGAGAAGCACCTGCAC-3′ and RV, 5′-GGCCGCCCGGGAATTCCTAAGGAGTCCTCAATCCC-3′. The pBacPAK-His3-GSTPKCι kinase domain construct was as previously described (Kjaer et al., 2013).

### Transfections, immunoprecipitation and protein purification

HCT116 cells were transiently transfected with cDNA using FuGENE HD transfection reagent (Promega) according to the manufacturer's instructions. Caco2 cells were transiently transfected with cDNA using Lipofectamine LTX with Plus reagent (Thermo Fisher Scientific) according to the manufacturer's instructions. For siRNA transfections, HCT116 cells were reverse transfected with siRNA using HiPerFect siRNA transfection reagent (Qiagen). siRNA was used at 20 nM unless otherwise stated. Caco2 cells were reverse transfected with siRNA using Lipofectamine RNAiMAX (Thermo Fisher Scientific). siRNA was used at 40 nM unless stated otherwise.

GFP-traps and FLAG immunoprecipitations were performed using GFP-Trap-M magnetic beads (Chromotek) and FLAG-M2 beads (Sigma); FLAG immunoprecipitations were performed using FLAG-M2 magnetic agarose resin (Sigma), and uncoupled magnetic particles (Chromotek) were used for pre-clearing. Co-immunoprecipitation was typically performed at 48 h after cDNA transfection and 72 h after siRNA transfection. Cells were lysed in lysis buffer [20 mM Tris-HCl pH 8, 130 mM NaCl, 1% (w/v) Triton X-100,1 mM DTT, 10 mM NaF with added protease inhibitor cocktail (CoMplete, Roche) and phosphatase inhibitor cocktail set II and set IV (Merck Millipore)]. Cell lysates were centrifuged at 16,000 ***g*** in a table top centrifuge for 10 min at 4°C. The supernatant was pre-cleared by incubation with magnetic particles (Chromotek) at 4°C for 1 h while rotating. Pre-cleared lysates were incubated with beads at 4°C for 90 min on a rotating wheel. Beads were then washed five times with co-IP wash buffer followed by elution with 2× Laemmli sample buffer. Co-immunoprecipitation samples were boiled at 95°C for 5 min and analysed by SDS-PAGE and immunoblotting.

### Co-expression of FARP2-FERM-FA and aPKCι kinase domain followed by size-exclusion chromatography

Viruses encoding His–FARP2 FERM-FA and the GST–His-tagged PKCι kinase domain were used to infect 50 ml cultures of Sf21 cells at 1×106 cells/ml [multiplicity of infection (MOI)=1]. Cultures were allowed to grow for three days after which the cells were harvested and lysed. GST–His-PKCι kinase domain – His–FARP2 FERM-FA complexes were purified from Sf21 cultures using glutathione–Sepharose. Complexes were eluted from the resin by 3C protease cleavage (Francis Crick Institute, Science Technology Platform), and the eluates was loaded on an S200 increase size exclusion column (GE Healthcare). Elution fractions were analysed by SDS-PAGE.

### Ca switch and TER

A junction formation assay based on a Ca^2+^ switch was performed as previously described and processed for either immunofluorescence or TER (Elbediwy et al., 2012). Cell maintenance TER measurements were performed in normal medium. Briefly cells were reverse transfected, and left for 24 h before being reseeded in transwells. TER was assessed at 48 h post transfection and the protocol used was as previously described (Elbediwy et al., 2012).

### G-LISA

Cdc42 and Rac1/2/3 activity was assessed using a colorimetric based G-LISA activation assay kit (Cytoskeleton). On day 1, Caco2 cells were reverse transfected with 80 nM of siRNA. On day 4 (72 h post transfection), cells were washed on ice with cold PBS and processed according to the manufacturer's instructions (Elbediwy et al., 2012).

### FRET-FLIM

FLIM microscopy was performed using a Leica SP8 confocal microscope. GFP–PKCι was excited with a 488 nm pulsed laser excitation and, for samples co-transfected with FARP2–FLAG and labelled with an anti-HA antibody conjugated to Alexa Fluor 647 (Cell Signaling), a 640 nm laser was used to collect a standard confocal image. Using FLIMfit (Warren et al., 2013) to analyse the lifetime decays, data from 28 different cells contributed to the donor-only histogram and 80 cells contributed to the donor-plus-acceptor histogram. Regions of interest corresponding to cell membranes were drawn by hand and the values of the calculated lifetimes for these pixels were combined into intensity weighted histograms of donor-only or donor-plus-acceptor lifetimes. These distributions were converted into distributions of FRET efficiency.

### Quantification

Quantification of continuous staining for ZO-1, aPKC or transfected FARP WT in Caco-2 cells was scored as either a continuous stain, in which the junctional antibody staining formed a complete ring around the cell, or discontinuous, in which the junctional antibody staining was repeatedly broken or fragmented around the cell. Cells were assessed over three independent experiments counting 50–200 transfected cells for each condition or 200–500 non-transfected cells. This method was described previously (Aguilar-Aragon et al., 2018; Elbediwy et al., 2012). For graph production and statistical significance, Prism was used and the software automatically calculated the statistical significance using a Student's *t*-test. Error bars represent the s.d. Significance as illustrated by the presence of asterisks is as follows: ns, not significant (*P*>0.05); *P≤0.05; **P≤0.01; ***P≤0.001; ****P≤0.0001.

## Supplementary Material

Supplementary information
