# Supplementary material for: The Rho family GEF FARP2 is activated by aPKCι to control tight junction formation and polarity
Source: J Cell Sci. 2019 Apr 25;132(8):jcs223743. doi: 10.1242/jcs.223743 (PMC6503954; doi:10.1242/jcs.223743)
Supplement: Supplementary information [file joces-132-223743-s1.pdf]

FIGURE S1

A

	Tryptic fragments (#)		
Identified Protein	Exp 1	Exp2	Exp3
PKCι (WT)	36	23	46
FARP2	2	0	5

B

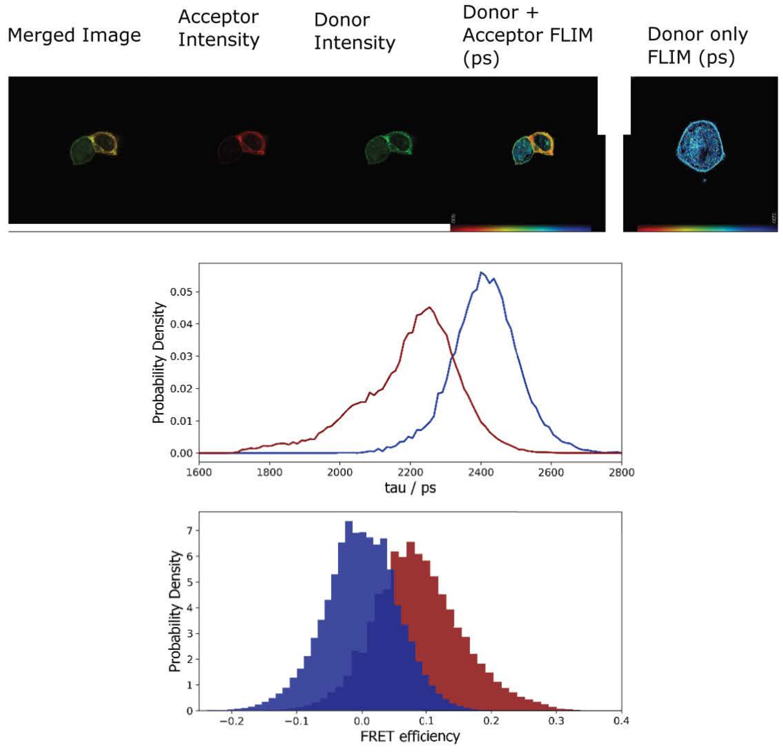

C

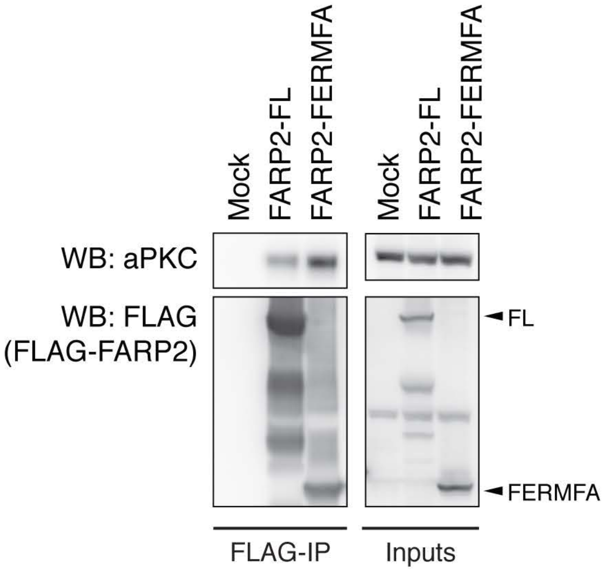

D

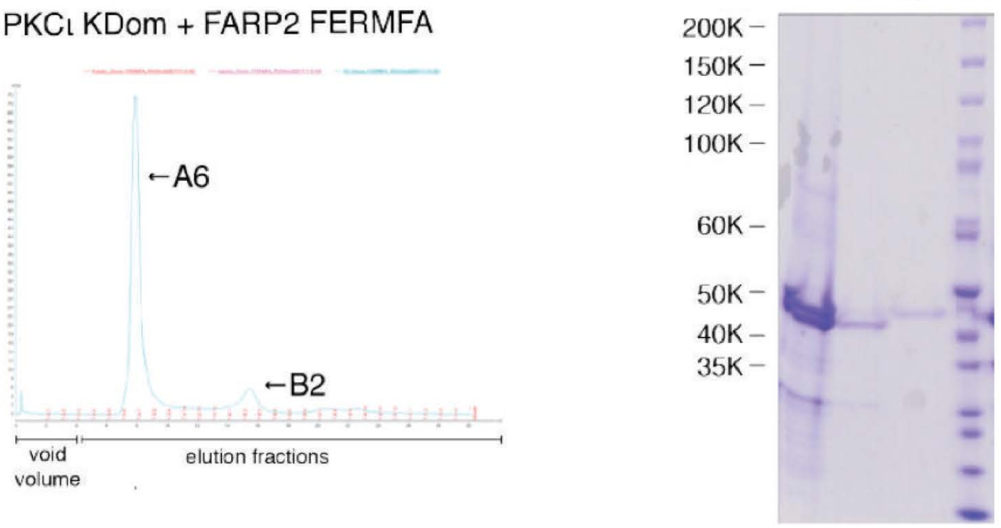

E

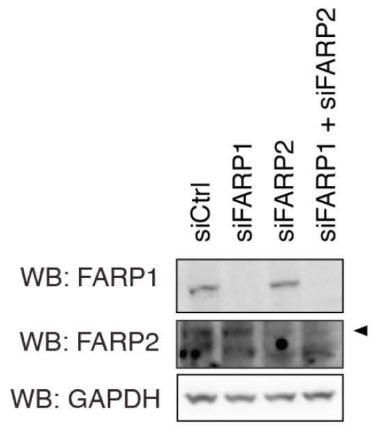

Journal of Cell Science • Supplementary information

FIGURE S1

A. FARP2 is discovered as a novel interactor of aPKCι in IP-MS experiments. The Table displays the number of tryptic fragments identified in IP-MS for GFP-tagged aPKCι for either aPKCι or FARP2. B. GFP-PKCι and FLAG-FARP2 were coexpressed in HCT116 cells. GFP lifetime was monitored at 488nm in the absence (indicated Donor only) or presence of Anti-HA Alexa 647 as exemplified in the upper panels. Lifetime values of doubly transfected cells were captured and quantified as described in the materials and methods. Lifetimes (tau) in picoseconds (ps) and the derived FRET efficiencies are shown for the donor only (blue) and donor-acceptor (umber) analyses. C. aPKCι interacts with FARP2 through its FERM-FA domains. HCT116 cells were co-transfected with plasmids expressing FLAG-tagged FARP and GFP or GFP-tagged aPKCι; immunoprecipitates were analysed with the indicated antibodies. D. GST-PKCι kinase domain was co-expressed with the FARP2 FERM-FA domain in sf9 cells. The kinase was purified on glutathione Sepharose beads and the purified material analysed by SDS-PAGE and Coomassie staining (Lane 1). The 3C cleaved products eluted from the beads were gel filtered (see left panel) and the two peaks identified were subjected to SDS-PAGE. The separated proteins are evident in lanes 2 and 3. E. analysis of FARP1/2 knockdown in CaCo2 cells via western blot. This figure refers to the experiment in Fig. 1D.

FIGURE S2

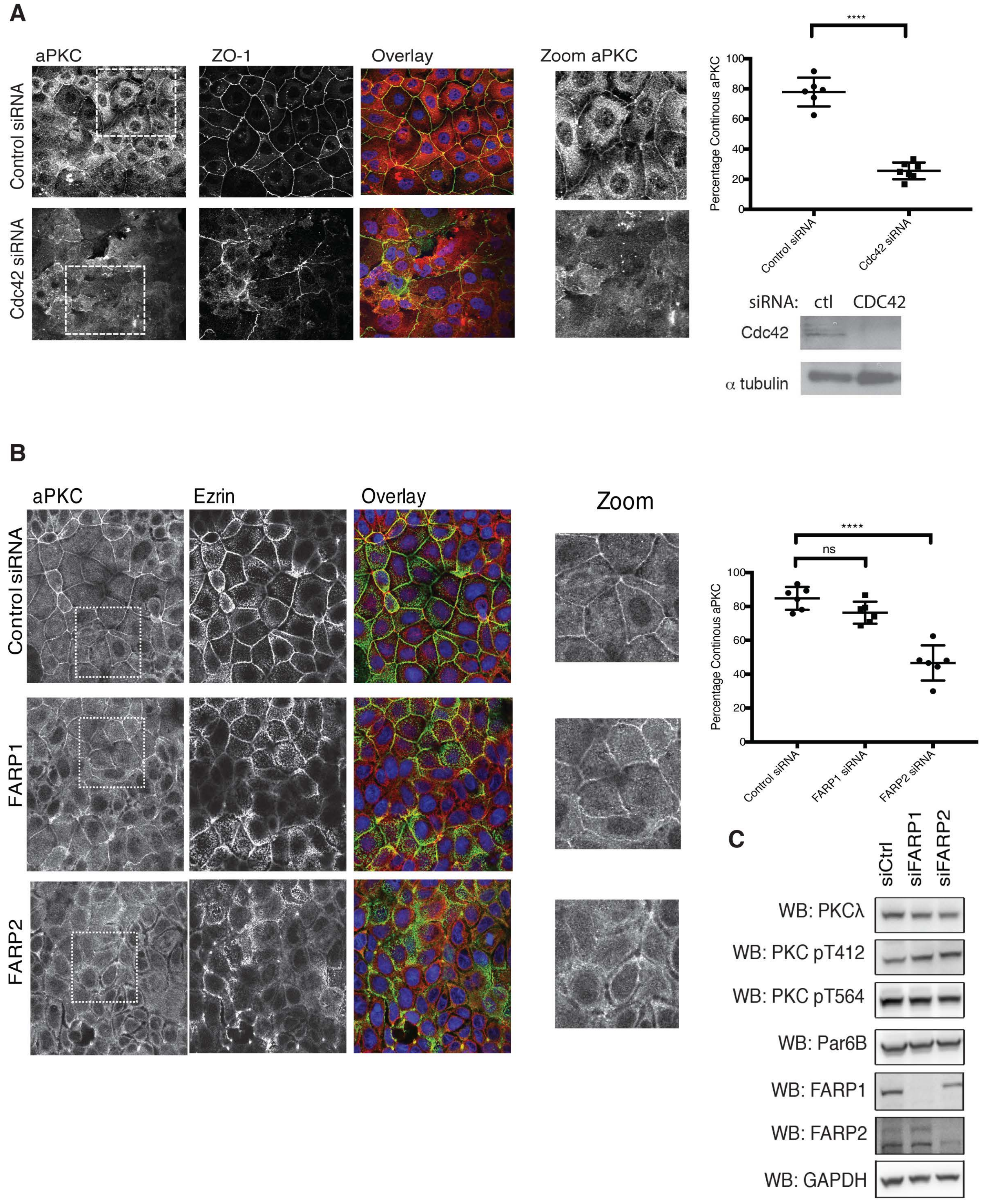

Journal of Cell Science • Supplementary information

FIGURE S2

A. siRNA of Cdc42 phenocopies the effect of FARP2 depletion on the polarity marker ZO-1. Western blot shows efficiency of knockdown. Representative example, n=3 independent experiments with each experiment containing n=6 samples. Quantification as described in Materials and Methods is shown on the right; ****: $p \leq 0.0001$. B. immunofluorescence showing the effect of RNAi on polarity markers when depleting FARP1 and FARP2. Note that the polarity marker Ezrin and aPKC are unaffected when depleting FARP1, while localisation of both Ezrin and aPKC is severely perturbed upon siRNA of FARP2. Representative example, n=3 independent experiments with each experiment containing n=6 samples. Quantification as described in Materials and Methods is shown on the right; ns: not significant ****: $p \leq 0.0001$ C. Immunoblot of FARP1 and FARP2 shows no effect on the expression levels of aPKC priming sites and the polarity marker PAR6 suggesting the effect on aPKC seen in A is a localisation effect rather than a degradation/expression effect.

FIGURE S3

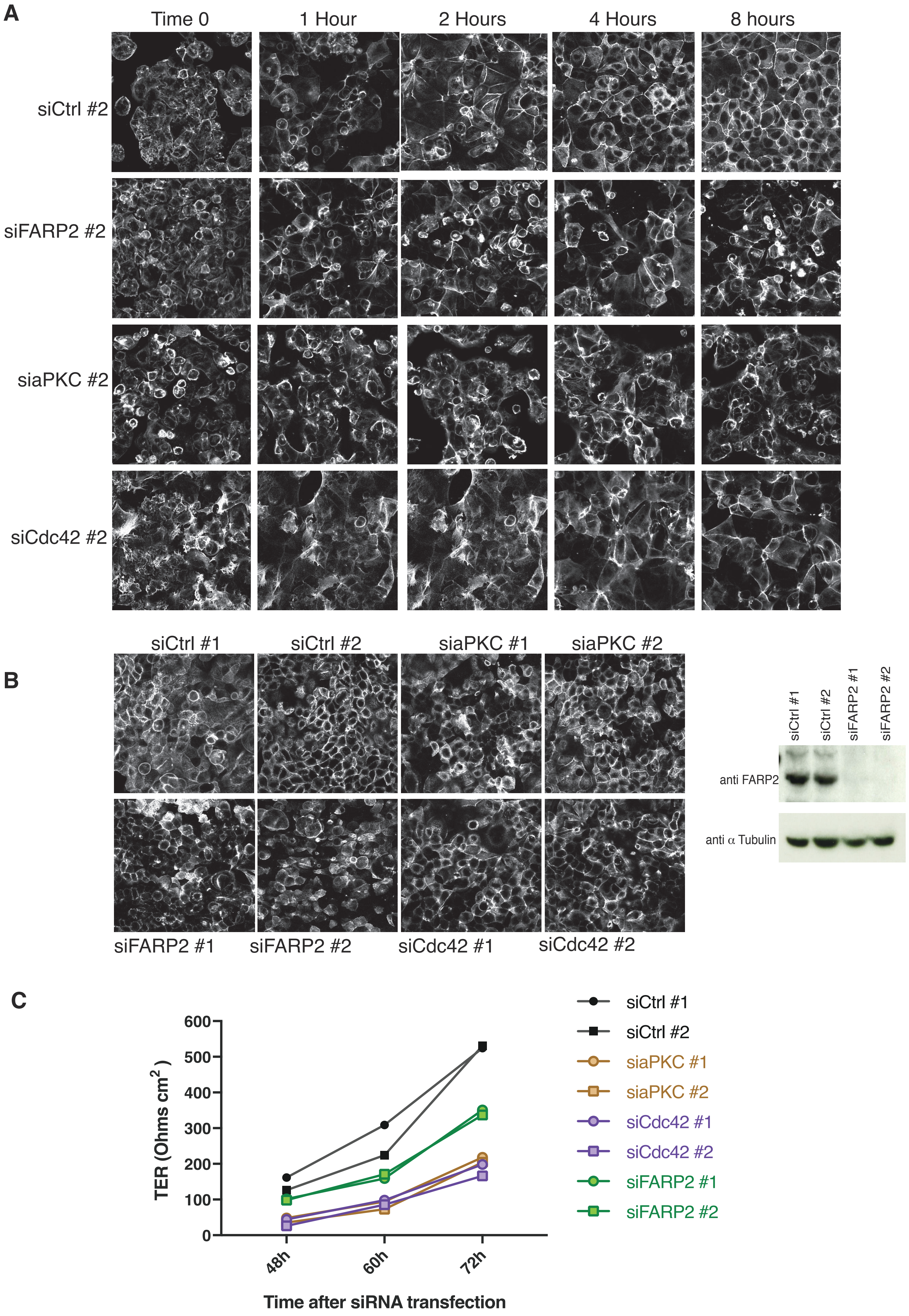

Journal of Cell Science • Supplementary information

FIGURE S3

A. FARP2 siRNA deconvolution using a second siRNA oligo causes severe disruption of the junctional marker ZO-1 during junction establishment in a calcium switch. Representative example, n=3 independent experiments with each experiment containing n=6 samples. B. FARP2 siRNA deconvolution using two separate oligos shows the junctional impairment of the marker ZO-1 during RNAi in steady state. A corresponding western blot shows the knockdown efficiency of the FARP2 siRNA Representative example, n=2 independent experiments with each experiment containing n=6 samples. C. Deconvolution of FARP2, aPKC and Cdc42 RNAi single oligos during a steady state maintenance Trans-epithelial resistance assay shows impairment of the junctions. Representative example, n=2 independent experiments with each experiment containing n=6 samples.

FIGURE S4

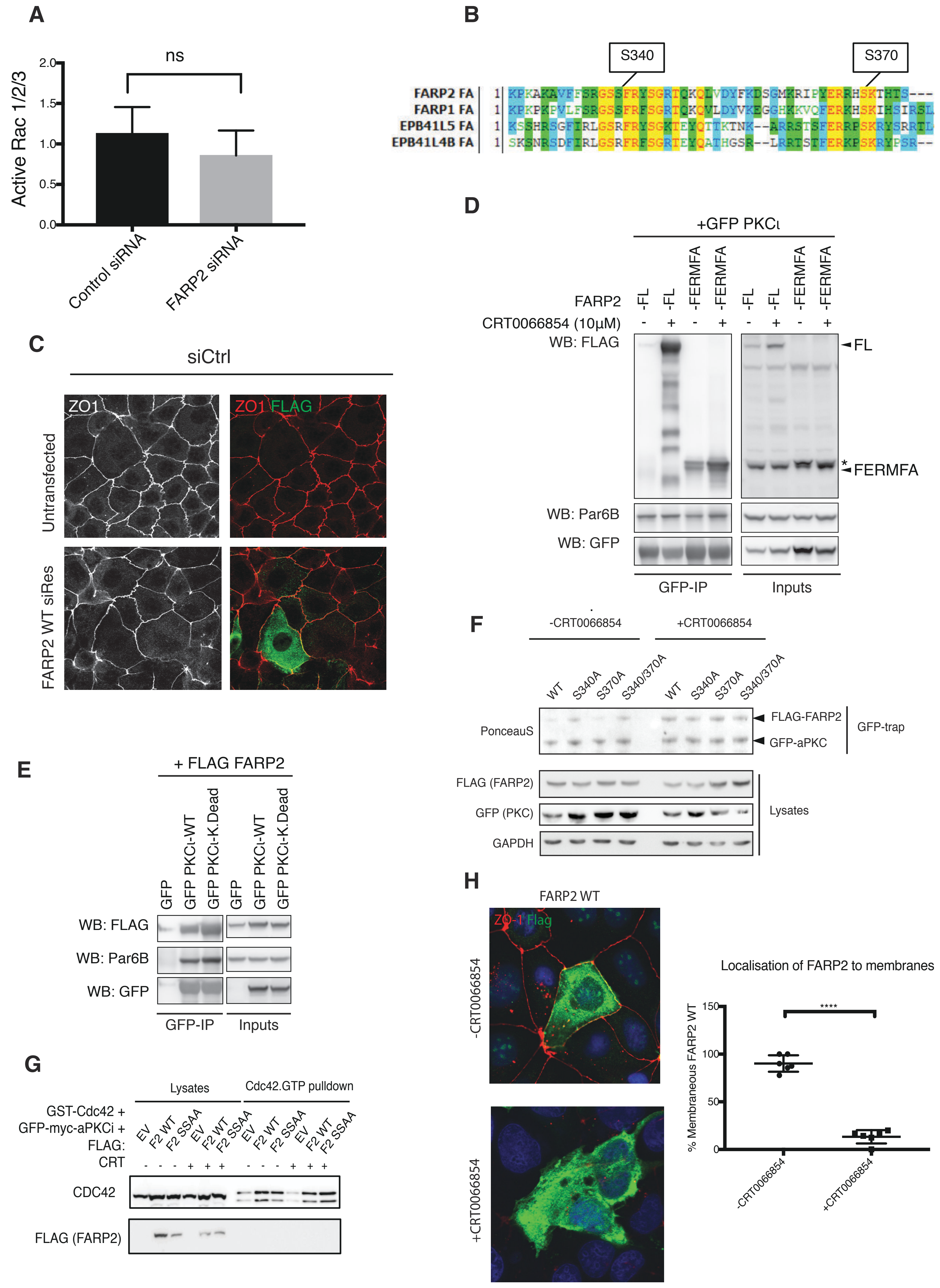

Journal of Cell Science • Supplementary information

FIGURE S4

A. GLISA assay assessing the levels of active (GTP-bound) Rac1/2/3 in CaCo2 cells transfected with either control siRNA or siRNA targeting FARP2. Knockdown of FARP2 does not result in a significant reduction of active Rac-levels, indicating its specific action on Cdc42 (Fig 3A). Representative example, n=3 independent experiments with each experiment containing n=4 samples. .

B. Alignment of the FA domain of FERM-FA containing proteins shows conservation of aPKCι target sites. FARP2 Ser-370 is conserved in all four FERM-FA containing proteins, whereas Ser-340 is present specifically in FARP1/2. C. Expression of siRNA resistant FARP2 in Control siRNA conditions. Cells were transfected with scrambled non-targeting siRNA and subsequently with an siRNA-resistant FLAG-FARP2 cDNA. An illustrative IF image is shown indicating a FLAG-positive, transfected cell. Representative example, n=3 independent experiments with each experiment containing n=6 samples.. D. Enhanced complex formation between FARP2 and inhibited aPKCι HCT116 cells were co-transfected with plasmids expressing FLAG-tagged FARP2 (full-length or the FERM-FA domain) and GFP-tagged WT aPKCι. Forty-eight hours post-transfection cells were treated with CRT0066854 (10 μM) or DMSO for 60 min. and immunoprecipitates were analysed with the indicated antibodies. There is an increased recovery of aPKCι associated with FARP2 upon aPKCι inhibition. Representative blots of three independent experiments are shown. E. FARP2 and WT or inactive aPKCι were co-transfected in HCT116 cells with plasmids expressing FLAG-tagged FARP2. aPKCι immunoprecipitates were analysed with the indicated antibodies. There is an increased association of FARP2 with kinase-dead aPKCι supporting the observations in Fig. S3D. Representative blots of three independent experiments are shown. F. As in Fig. S3D, PKCι was coexpressed with FARP2 and immunocomplexes were analysed by western blot as indicated. The FARP2 variants employed here were those with defective aPKCι phosphorylation sites, individually or in combination (see text). Increased recovery was observed in the mutant forms, particularly those carrying the S340A mutation. For comparison, equivalent levels of FARP2 were recovered for all FARP2 forms in the presence of the PKCι inhibitor CRT0066854. Representative blots of three independent experiments are shown. G. Western blot showing increased levels of active CDC42 upon ectopic expression of FARP2 WT or a S340/370A mutant. Active levels of CDC42 were measured using the Active Cdc42 Pull-Down and Detection Kit (Thermo Fisher Scientific) according to the manufacturers' instructions. Representative blots of two independent experiments are shown. H. Expression of Flag FARP2 wild type in presence of the aPKC inhibitor CRT0066854 phenocopies the FARP2 S340A/S370A mutant in the lack of rescue on the polarity component ZO-1. Representative example, n=2 independent experiments with each experiment containing n=6 samples. Quantification as described in Materials and Methods is shown on the right ****: $p \leq 0.0001$

Journal of Cell Science • Supplementary information
